# Absolute quantitation of microbiota abundance in environmental samples

**DOI:** 10.1186/s40168-018-0491-7

**Published:** 2018-06-19

**Authors:** Andrzej Tkacz, Marion Hortala, Philip S. Poole

**Affiliations:** Department of Plant Sciences, University of Oxford, South Parks Road, Oxford, OX1 3RB UK

## Abstract

**Background:**

Microbial communities (microbiota) influence human and animal disease and immunity, geochemical nutrient cycling and plant productivity. Specific groups, including bacteria, archaea, eukaryotes or fungi, are amplified by PCR to assess the relative abundance of sub-groups (e.g. genera). However, neither the absolute abundance of sub-groups is revealed, nor can different amplicon families (i.e. OTUs derived from a specific pair of PCR primers such as bacterial 16S, eukaryotic 18S or fungi ITS) be compared. This prevents determination of the absolute abundance of a particular group and domain-level shifts in microbiota abundance can remain undetected.

**Results:**

We have developed absolute quantitation of amplicon families using synthetic chimeric DNA spikes. Synthetic spikes were added directly to environmental samples, co-isolated and PCR-amplified, allowing calculation of the absolute abundance of amplicon families (e.g. prokaryotic 16S, eukaryotic 18S and fungal ITS per unit mass of sample).

**Conclusions:**

Spikes can be adapted to any amplicon-specific group including rhizobia from soils, Firmicutes and Bifidobacteria from human gut or Enterobacteriaceae from food samples. Crucially, using highly complex soil samples, we show that the absolute abundance of specific groups can remain steady or increase, even when their relative abundance decreases. Thus, without absolute quantitation, the underlying pathology, physiology and ecology of microbial groups may be masked by their relative abundance.

**Electronic supplementary material:**

The online version of this article (10.1186/s40168-018-0491-7) contains supplementary material, which is available to authorized users.

## Background

Over the last decade, affordable amplicon DNA sequencing has revealed that the microbiota influences human immunity [1], digestion [2], mental health [3], and plant growth and development [4]. These studies in diverse fields have revealed a delicate relationship among different microbial groups and, in the case of plants, have highlighted the importance of the microbial community of soil in plant health [5, 6]. The environmental microbial community has multiple components that are normally abundant and are typically investigated by PCR amplification of marker genes. However, for most studies, only one microbiota domain, such as bacteria, is investigated. Furthermore, most microbiota studies are limited by the production of PCR amplicons (amplified genes from an  individual environmental sample) using domain-specific primers. This in turn leads to loss of quantitative comparison between any two or more groups of PCR amplicons. However, in order to unravel the real complexity of the gut, soil and other environments, the quantitative relations between major microbial groups must be determined.

There is a complex relationship between interconnected bacterial, archaeal, fungal, viral and single-cell eukaryotic organisms, all of which are PCR-amplified to varying extents by different sets of PCR primers. Current DNA technologies allow parallel sequencing of multiple samples, yielding millions of short reads. The most common way to profile the prokaryotic community is to amplify the 16S rRNA gene, while profiling of eukaryotes is typically performed by 18S rRNA amplification. However, from PCR experiments using different primer pairs, any cross-domain comparison of microbiota is impossible. The problem of quantitative comparison becomes even more intractable when considering primers designed for fungi or specific groups of bacteria, such as Bifidobacteria, that are key components of the gut microbiota [7]. There are a few strategies attempting to overcome this problem. Gene-coding proteins, such as Cpn60, are universally present in prokaryotes and in eukaryotic mitochondria and chloroplasts. They can therefore be used to profile prokaryotic communities and compare their relative abundance against eukaryotic host organelle abundance [8]. Moreover, comparisons based on the gene-encoding Cpn60 proved to be useful at predicting bacterial genome similarities [9]. However, the current limitation of this method is a relatively small reference database. A set of life-universal primers was developed based on a similarity between prokaryotic and eukaryotic ribosomal gene sequences [10]. It is a promising advance in the field, but the primers were developed for sponge symbionts and may lose their phylogenetic compatibility in more complex gut or soil environments. Microbial profiling can also be made using housekeeping genes as *rpoB*, *amoA*, *pmoA*, *nirS*, *nirK*, *nosZ* and *pufM* [11]. Among these genes, *rpoB* was shown to be very powerful at discriminating closely related species [12]. Moreover, these approaches based on the gene encoding Cpn60 or universal 16S/18S rRNA genes, despite being able to compare the relative abundance within a domain, do not provide estimation of the total DNA abundance in a sample. qPCR can be used for the estimation of microbial abundance, as it allows calculation of the copy number of a specific gene per amount of total DNA. However, it does not provide information on the environmental in situ gene abundance. Furthermore, it requires separate and complex qPCR analysis for each group, subsequent to initial metagenomic sequencing. Other approaches include measurements based on tri-phosphate abundance (ATP), flow cytometry (FCM), phospholipid fatty acids (PLFA) and microbial biomass carbon (MBC) [13].

An elaborate flow cytometry method was used in a breakthrough study identifying microbiome abundance as a key driver in Crohn’s disease. Measurement of the absolute abundance of organisms revealed the ratio of Bacteroides to *Prevotella*, which is considered an important maker of gut health, is an artefact of relative quantification [14]. While such an elaborate method is not easily transferred to other organisms, it is possible to ‘spike’ samples, by adding a known number of bacterial cells of a species not normally found in a given environment. Mammalian gut samples were spiked with an extreme soil halophile, not present in gut, and the absolute abundance of all groups determined by comparison to the number of 16S rRNA reads of the halophile relative to its input abundance [15]. This is limited by the requirement for prior knowledge of which bacterial species are absent from an environment and the necessity of preparing a culture to a highly controlled cell density.

In RNA-seq-based studies, it is common to add synthetic RNA standard to an environmental sample prior to RNA isolation. Environmental and synthetic RNA is then co-purified, converted to cDNA, and sequenced. From the normalised ratio of these in the sequencing output, it is possible to compare RNA relative abundance between the samples [16, 17].

Here, we show results of a DNA-based PCR amplicon adaptation of the RNA-seq method. We have designed short chimeric synthetic DNA fragments that contain universal primer binding sites specific for three major microbiota domains: prokaryotes, eukaryotes and fungi. During PCR, these synthetic DNA molecules produce their respective expected amplicon size, due to the presence of a synthetic stuffer region. Adding a known amount of synthetic DNA spikes directly to environmental samples and calculating their relative abundance in the sequencing output allows the absolute abundance of specific groups of organisms to be determined both within and between amplicon classes. Here, we demonstrate the strength of this approach with both pure bacterial cultures and complex soil samples. We first test our spiking approach on a define number of bacterial cells. Later, we show how this method can be applied to samples with an unknown microbiota structure. According to different estimates, the soil microbiota is at least an order of magnitude more diverse than that of the gut [18]. Hence, this method can be adapted for analysis of simpler environments such as that of food samples or human/animal gut. Strikingly, we show that when comparing samples, the relative abundance of microbial taxa may be higher in a sample even when its absolute abundance is lower.

## Methods

### Design of P, E and F synthetic spikes

**Fig. 1:**
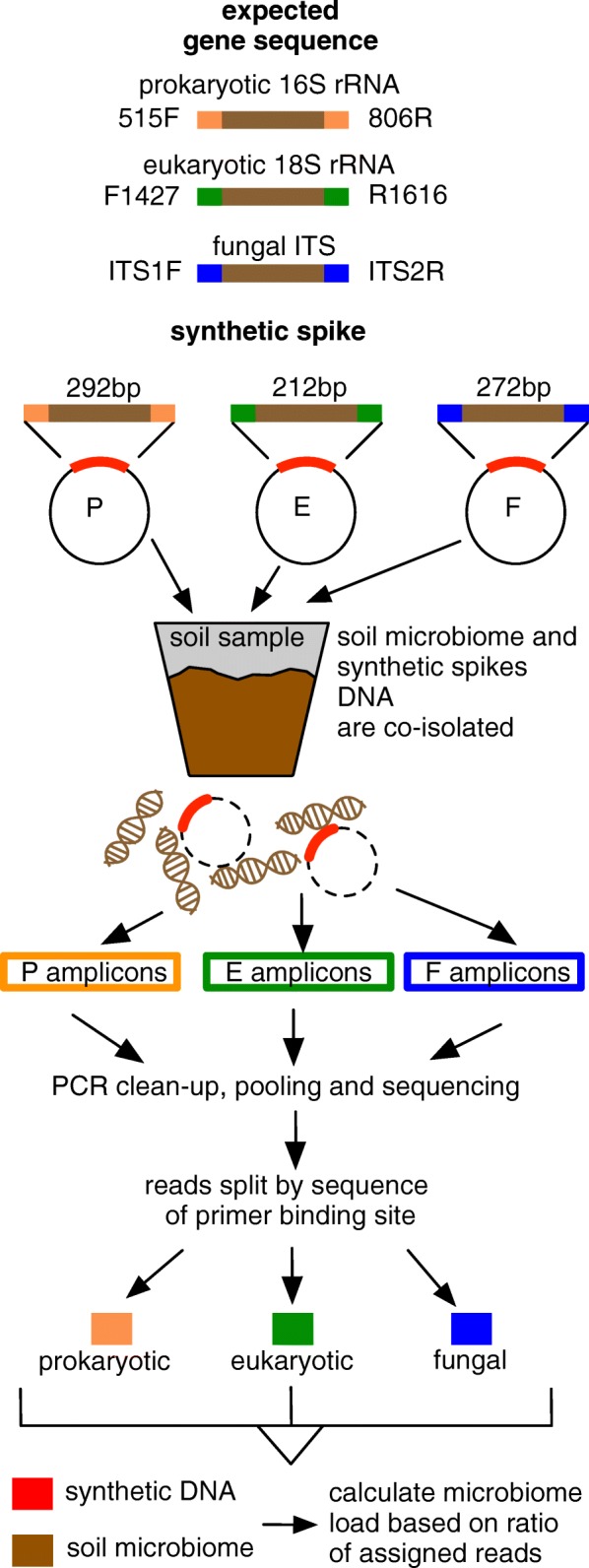
Synthetic spike design. P, E and F synthetic spikes were designed using PBS sequences, together with the length and GC content of amplicons from prokaryotes (P), eukaryotes (E) and fungi (F), respectively. For P synthetic spikes the primer binding sites (PBS) shown in orange, for E in green and for F in blue

Synthetic spikes were designed with three key elements: (i) primer binding sites (PBSs) from the common genes used for identification of prokaryotes (P), eukaryotes (E) and fungi (F), respectively; (ii) an optimised synthetic stuffer sequence of the same length and GC content as the in vivo target and (iii) a readily available, easy to handle source of synthetic spike DNA (Fig. 1).

PBSs are based on the three sets of PCR primers chosen for microbiota amplification. For prokaryotes (P), the prokaryotic 16S rRNA V4 region primers 515F and 806R [19] were used as they are commonly used in soil studies. For eukaryotes (E), we used the 18S primer pair, F1427 and R1616 [20], that targets a broad range of eukaryotic taxa including algae, diatoms, animals, excavates (protists, flagellates), fungi and moulds. The third primer pair specifically targets fungi (F), ITS1F and ITS2R and are widely used in soil microbiota studies [21]. These primers target the variable sized ITS fragment of Ascomycota and Basidiomycota, common phyla in forest soils [22]. As variability in amplicon length can bias PCR amplification efficiency, the length of the stuffer sequences of P (16S rRNA) and E (18S rRNA) were matched to the length of natural PCR products. Fungal ITS amplicons show more variability in length, but based on the results of our previous sequencing, the most common size was 272 bp [23]; therefore, a stuffer sequence of 272 bp was used in designing the F synthetic spike (Fig. 1). In each case, GC content was designed to be similar to their environmental gene counterparts with sequences designed using a random DNA generator (https://www.faculty.ucr.edu). P, E and F synthetic spikes were synthesised by Geneart (Invitrogen) and supplied cloned in plasmid pMA-T, forming pSpike-P, pSpike-E and pSpike-F, respectively. Plasmid were transformed into *Escherichia coli* and deposited at https://www.addgene.com as plasmids #101172, #101173 and #101174.

### Characterisation of soil samples

The two soils used come from fallow ground, which has not been cultivated for at least 20 years. Bawburgh soil with NO_3_^−^3.49 mg kg^−1^, P^−3^ 120.5 mg kg^− 1^, K^+^ 168.2 mg kg^−1^, Mg^2+^ 33.55 mg kg^−1^ and containing relatively low organic matter content 2.92%, pH 7.5 was characterised previously [24]. Wytham soil comes from Wytham Woods, University of Oxford and was collected from a forest opening at 51**°**46′14′′N and 1**°**20′18′′W. It was chemically characterised (Hutton-Analytical, James Hutton Limited, Aberdeen) and shown to contain P^−3^ 122.9 mg kg^−1^, K^+^ 483.6 mg kg^−1^ and Mg^2+^ 304.9 mg kg^−1^, organic matter 16.78%, pH 7.22. Wytham gleysol is significantly richer in minerals and organic matter than the luvisol of Bawburgh; however, they have a similar pH. Both soils were air-dried prior to analysis in order to minimise differences between their weight and volume.

### Microbiological techniques:

*Rhizobium leguminosarum* bv. *viciae* 3841 cultures (100 ml) were grown in TY [25], a rich growth medium until OD_600_ of 0.54, spun down and re-suspended in 50 ml of minimal media [26] to stop bacterial growth. Bacterial counts using serial dilution assay were performed to assess bacterial numbers.

The abundance *of Rhizobium* cells was measured by optical density (OD_600_) and plate counting. 1.11 × 10^9^ colony forming units (cfu) per 1 ml of bacterial suspension was used in each experiment. When DNA was isolated prior to the addition of synthetic spikes, the data was corrected for DNA loss during column-based isolation (predicted total amount of DNA divided by amount of DNA mixed with synthetic spikes and used for PCR).

**Fig. 2:**
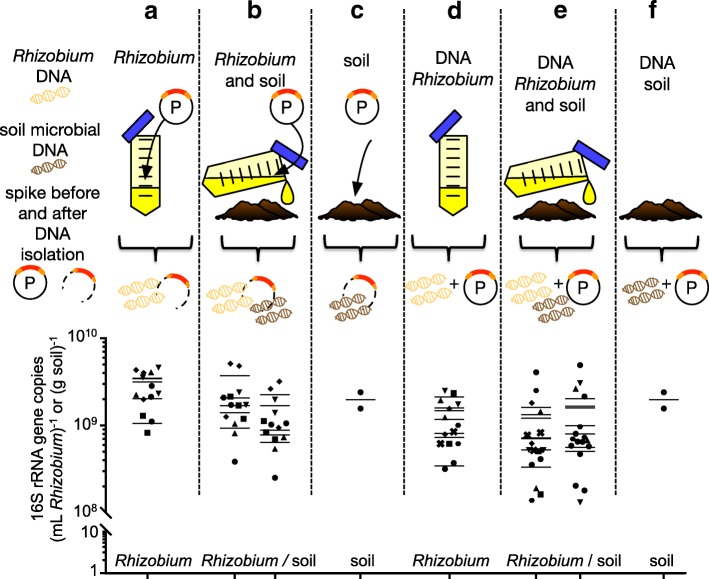
Effect of added spikes before and after DNA isolation on microbial quantitation. P synthetic spike DNA was added to samples prior to DNA isolation (**a**–**c**) in a concentration gradient (**a**–**b**) of 14 (circle), 34 (square), 70 (upward triangle), 140 (downward triangle) and 340 (diamond) pg per *Rhizobium* culture (1 ml) or soil (1 g) and (**c**) 39 (square) pg per soil (1 g) and then co-isolated with microbial DNA. Spikes were also added to purified microbial DNA (**d**–**f**) in a concentration gradient of 2 (circle), 4 (square), 8 (upward triangle), 20 (downward triangle), 40 (diamond), 80 (hexagon) and 200 (cross) pg per isolation microbial DNA (μg). **a**–**c** The synthetic spike plasmid DNA may be cleaved during DNA purification. Horizontal lines represent the average no of 16S rRNA for each group of samples. Bawburgh soil was used throughout

For experiments with *Rhizobium* cells added to soil, prior to DNA isolation (Fig. 2a–c), P synthetic spike was added using a concentration gradient of 14 to 340 pg of spikes per 1 ml of *Rhizobium* or 1 g of soil. For experiments with DNA isolated from *Rhizobium* added to Bawburgh soil (Fig. 2d–f), P synthetic spike was added to previously isolated DNA at seven different concentrations: 2, 4, 8, 20, 40, 80 and 200 pg per 1 μg of microbial DNA.

In experiments comparing Bawburgh and Wytham soil, 300 mg of soil was routinely used. Even though the soil was well mixed, some variation in the microbial composition and abundance would be expected. For each soil, 24 samples were prepared, with the eight levels of PEF synthetic spikes used (Table 1) performed in triplicate. These levels contain a defined amount of each of the P, E and F synthetic spikes. We anticipated a decreased abundance of eukaryotic 18S rRNA and fungal ITS compared with prokaryotic 16S rRNA based on our previous work [27], and hence, we used different amounts of PEF spikes.

**Table 1:** Effect of the level of P, E and F synthetic spikes. (pg DNA) added to Bawburgh and Wytham soil (1 g)

Synthetic spike level	Amount relative to level 8 (%)	P synthetic spike (pg)	Copies of 16S rRNA added	E synthetic spike (pg)	Copies of 18S rRNA added	F synthetic spike (pg)	Copies of ITS added
1	1	97	3.5E + 07	57	2.1E + 07	32	1.2E + 07
2	2	193	7.1E + 07	113	4.3E + 07	64	2.4E + 07
3	5	483	1.8E + 08	283	1.1E + 08	161	5.9E + 07
4	10	966	3.5E + 08	566	2.1E + 08	321	1.2E + 08
5	15	1449	5.3E + 08	849	3.2E + 08	482	1.8E + 08
6	20	1931	7.1E + 08	1132	4.3E + 08	643	2.4E + 08
7	50	4829	1.8E + 09	2831	1.1E + 09	1607	5.9E + 08
8	100	9657	3.5E + 09	5661	2.1E + 09	3213	1.2E + 09
Spike (1 ng) contains			3.7E + 08		3.8E + 08		3.7E + 08

Samples were processed using a soil DNA isolation kit (D6001, Zymo research, Irvine, US) according to the manufacturer’s instructions. Microbial and synthetic spike DNA was co-isolated, co-amplified by PCR and co-sequenced. All the DNA concentration measurements were done using dsDNA BR Qubit kit. (Invitrogen). All reads were annotated using gene-specific databases, supplemented with P, E and F synthetic spike DNA sequences. Reads were annotated at the prokaryotic phyla, eukaryotic domain/phyla or division level, and fungal ITS reads were annotated at the genus and division level. The combined number of microbial-origin reads was compared with the number of reads that were attributed to each synthetic spike. In order to remove sample-to-sample variability caused by different sequencing depth, we calculated the relative abundance of synthetic spike reads per 1000 total reads.

### PCR, sequencing and qPCR

All primers are listed in Additional file 1.

In order to run domain-specific PCRs, samples were split into three aliquots and each amplified with a specific primer pair: either 515F/806R (for prokaryotes), F1427/R1616 (for eukaryotes) or ITSF1/ITSF2 (for fungi). A 2-step PCR system (DI, double indexing) was used as previously described [28]. Initial primers were not barcoded but contain 12 bp amplification ‘pads’ of known sequence at their 5′ end.

The first PCR amplifies the target gene and adds 12 bp pads on both sides of the amplicon. PCR conditions were as follows: high-fidelity Phusion (0.2 μl), HF buffer (4 μl) (Thermo Scientific F520 L), dNTPs (0.4 μl), primers (for each one 1 μl of 10 μM), template DNA (1.5 μl of 5 ng/μl) and H_2_O (11.9 μl) for each reaction (20 μl). PCR cycles were as follows: 98 °C for 1 min, 35 cycles of 98 °C for 15 s, 57 °C for 15 s and 72 °C for 30 s with a final elongation step of 72 °C for 7 min. PCR products were purified using a PCR clean-up kit (D4014, Zymo research) and used as DNA template for a second round of PCR. At this step, the amplification was performed using dual-barcoded primers targeting the 12 bp pads flanking the DNA template. PCR conditions were the same, with that the exception the cycle number was reduced to 25 and the annealing temperature increased to 61 °C. These parameters were chosen based on annealing temperature gradient PCR tests. Final PCR products were pooled and purified with the PCR clean-up kit. Samples were sent to MR DNA laboratory (Molecular Research LP, Texas, UK) for sequencing by an Illumina Miseq 300PE. Sequencing data was processed using custom-made Linux and Python scripts [23], supplemented with scripts from FASTX-toolkit (http://www.hannonlab.cshl.edu).

Quantitative PCR was performed using iQ SYBR Green Supermix (BioRad), template DNA (5 ng) and previously used primers for bacterial 16S rRNA and eukaryotic 18S rRNA genes. Reactions were incubated in a thermocycler (CFX96, BioRad) for 5 min at 95 °C, then 40 cycles of 15 s at 94 °C, 30 s at 57 °C (16S) or 64 °C (18S), and 30 s at 72 °C. Three technical replicates showed high reproducibility (mean SEM < 0.6% of mean), so only biological replicates (*n* = 10) were run. The proportions of bacteria and eukaryotes were calculated based on their Ct values.

### Data analysis

For each experiment ~ 1 M paired-end reads were aligned and binned according to the PBS and barcode sequences (Additional file 1). Reads were binned using zero-radius OTUs according to the Usearch10 pipeline with unoise3 algorithm [29] and annotated using the curated SILVA, PR2 and ITSone databases for prokaryotic 16S rRNA, eukaryotic 18S rRNA and fungal ITS, respectively. The DNA sequences of the PEF synthetic spikes were added to the databases.

Based on the annotation, reads were assigned into synthetic and microbial (domain/phyla level), or left unassigned as singleton OTUs or as reads of incorrect size, consisting of chimeras and sequencing errors. Only reads of 252–254 bp for 16S rRNA (99.4% of the reads), 210–212 bp for 18S rRNA (93.4% of the reads) and 250–300 bp for ITS amplicons (98.4% of the reads) were analysed (Additional file 2).

We removed samples with a low sequencing depth (less than 40% of the read number average per sample group), as well as samples for which results were an order of magnitude different from the other biological replicates. For these reasons, five samples for prokaryotes and four for eukaryotes were removed. We assume that this may be caused by either pipetting or PCR errors, especially in the first cycles of the process. In addition, eight prokaryotic samples were removed due to high laboratory *E. coli* contamination (Additional file 3). For clarity, we present the results obtained using the full dataset in Additional file 3 and Additional file 4: Figure S1 and Additional file 5: Figure S2.

In the culture control experiment, a total of 15 from 78 samples were removed as they showed a ratio of synthetic to microbial reads at least 2.5 times higher than the average, (calculation based on three biological replicates) (Additional file 1).

### Multidimensional scaling plots (MDS)

Phylogenetic data using zero-radius OTUs (without PEF synthetic spikes and singletons) was standardised, transformed by taking the square root and analysed using Bray-Curtis dissimilarity matrix visualised on MDS plots using PRIMER 6 software (PRIMER-E, Plymouth). This was to investigate whether addition of PEF synthetic spike DNA introduces any bias to microbial phylogenetic structure and to visually represent differences between two different soil microbiotas.

## Results

### Chimeric spiking gives an accurate estimation of microbial abundance

The accuracy of quantitative metagenomics was tested using a series of sampling strategies with known and unknown amounts of microbial DNA (Fig. 2).

P synthetic spikes were mixed using different proportions with 1.11 × 10^9^ cells of *Rhizobium*, and their DNA was isolated and co-amplified (Fig. 2a). Since the genome of this species of *Rhizobium* has three copies of the 16S rRNA gene, the expected outcome is 3.33 × 10^9^ 16S rRNA copies per 1 ml of culture. At the three highest levels of P spikes, the mean of the estimated *Rhizobium* gene content (3.11 × 10^9^, 3.44 × 10^9^ and 3.38 × 10^9^ 16S copies per 1 ml culture) was 99.3% of the actual amount (Fig. 2a). The lower spike levels appeared insufficient as they underestimated *Rhizobium* abundance (2.00 × 10^9^, 1.04 × 10^9^ 16S copies per 1 ml culture).

Furthermore, to check whether this might be changed by the presence of soil, P spikes were added to a culture of *Rhizobium* already mixed with soil (Fig. 2a). It can be seen that the estimation for *Rhizobium* rRNA copy number are more variable: 1.38 × 10^9^, 1.66 × 10^9^, 9.19 × 10^8^, 2.04 × 10^9^ and 3.67 × 10^9^ 16S rRNA copies per 1 ml of culture Fig. 2b). However, the fact that they are similar to the results obtained from pure cultures (Fig. 2a) indicates that addition of soil does not strongly interfere with the P synthetic spike soil DNA co-isolation and co-amplification by PCR. Potentially, soil particles could absorb some of the *Rhizobium*, it contains humic acids that may interfere with polymerase during PCR and it introduces a diverse microbial community, which makes the sequencing more difficult. It is likely some *Rhizobium* cells attached to soil particles, and their DNA was then not isolated completely. In this experiment, the soil microbiota was estimated to contain: 7.6 × 10^8^, 8.7 × 10^8^, 6.3 × 10^8^, 1.7 × 10^9^ and 2.2 × 10^9^ 16S rRNA copies per gram of soil. A similar value of 1.1 × 10^9^ 16S copies was estimated from a separate assay where P spikes were added to the soil alone (Fig. 2c). Hence, the spiking approach allowed for repeated estimation of the soil microbiota abundance with and without addition of a substantial amount of *Rhizobium* cells.

Next, spikes were added to already isolated bacterial and/or soil DNA (Fig. 2d–f). Addition of P synthetic spike to already isolated microbial DNA resulted in lower estimation of 16S rRNA gene copies. DNA from cultured *Rhizobium* gave an average estimate of 1.16 × 10^9^ 16S rRNA copies per 1 ml of culture (Fig. 2d) and DNA from cultured *Rhizobium* added to soil gave 9.2 × 10^8^ of *Rhizobium* 16S copies per 1 ml of culture (Fig. 2e). DNA from soil gave an average estimate of 16S rRNA gene copies of 1.17 × 10^9^ per 1 ml of culture, when measured mixed with *Rhizobium* culture and 1.96 × 10^9^ 16S copies per 1 ml of culture, when measured on its own (Fig. 2f). In order to obtain these values, the concentration of isolated DNA was measured to enable calculation of the abundance of *Rhizobium* and/or soil microbiota 16S rRNA copies. This may introduce a significant bias from estimation of the DNA quantity. Moreover, while the copy number of 16S rRNA is known for a defined species such as *Rhizobium*, it can only be approximated for unknown OTUs in the total microbiota. For comparative purposes the *Rhizobium* values were used, with a genome 7.8 Mb weighting 4 billion Da and 3 rRNA operon copy per genome, resulting in 377,155 16S rRNA gene copies per 1 ng of DNA. Based on these results, the most accurate results are obtained when spikes are added to the raw material prior to DNA isolation.

### Synthetic spikes allow measurement of in situ soil DNA abundance and microbiota composition in different soils

**Fig. 3:**
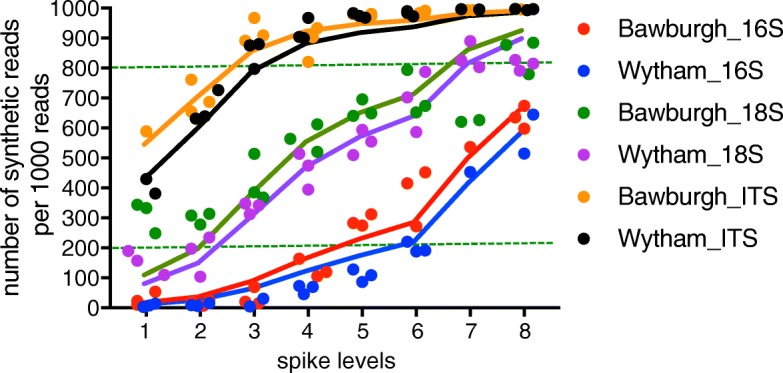
Model of synthetic spike addition. Number of sequencing reads of synthetic spike per 1000 total reads (*y-*axis) from 16S rRNA, 18S rRNA and ITS following addition to soil of different levels of P, E and F synthetic spikes (*x*-axis). Experimental results are shown by solid symbols while model data is presented with lines of corresponding colours. The model shows the expected spike contribution in the sequencing output for each spike level using the averaged gene abundance for a specific soil (as in Table 2). Dotted green lines indicate the region with 200–800 synthetic reads per 1000 reads. Spike levels on the *x*-axis correspond to the synthetic spike levels in Table 1

As addition of spikes allows accurate estimation of the abundance of the soil microbiota, total prokaryotic, eukaryotic and fungal abundances were measured in two contrasting soils. In order to estimate soil microbiota abundance, P (bacterial 16S rRNA), E (eukaryotic 18S) rRNA and F synthetic spikes (fungal ITS) were added to Bawburgh and Wytham soils (Table 1). A gradient of P, E and F spikes was used to test DNA isolation and/or PCR amplification bias between the amount of synthetic and environmental DNA. As expected, the higher the amount of synthetic spike added to soil samples, the higher their recovery in the sequencing output, although this plateaus at the highest levels of F synthetic spike (Fig. 3). In order to determine the optimum spike level, a simple model was constructed showing the expected number of synthetic sequences per 1000 total sequences. First, the microbial gene abundance was averaged (Fig. 3), and this value was used for modelling using the following equation:

$$ {\mathrm{SR}}_{1000}=\frac{\mathrm{NS}}{\left(\mathrm{NS}+\mathrm{ENV}\right)/1000} $$

where SR_1000_ is the number of synthetic reads per 1000 reads of the sequencing output. NS is the number of P, E or F spikes added per gram of soil. ENV is the gene copy number of prokaryotic 16S rRNA, eukaryotic 18S rRNA or fungal ITS per gram of soil.

**Fig. 4:**
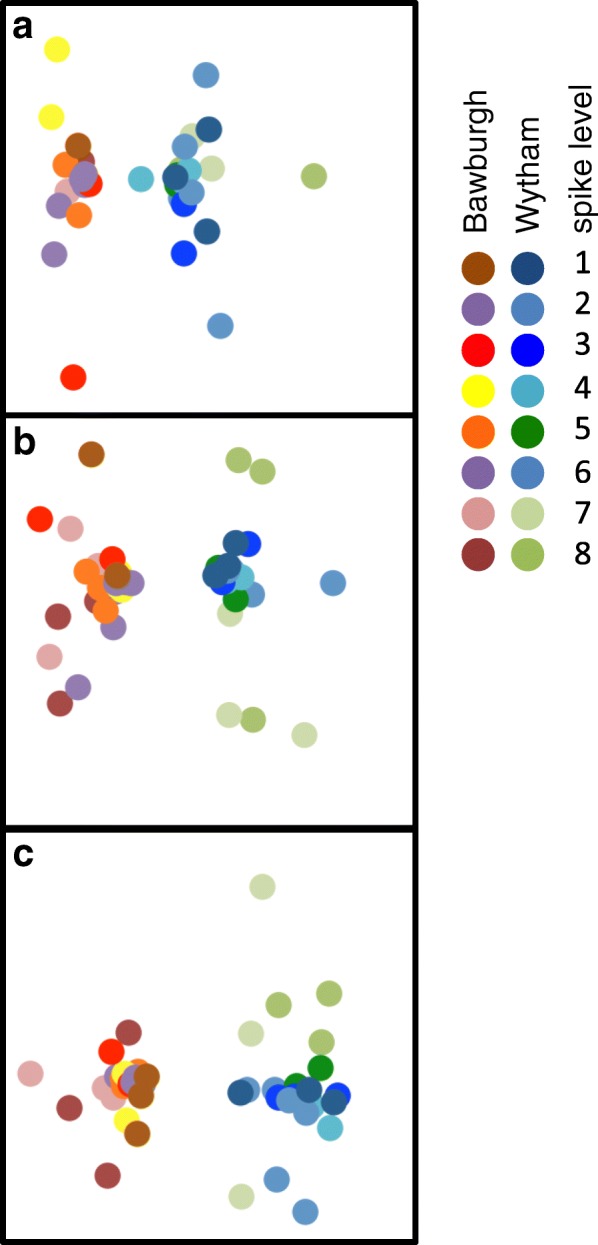
MDS plots of microbial community structure. **a** Prokaryotic **b** Eukaryotic and **c** fungal community. The microbial community of Bawburgh (warm colours) and Wytham (cool colours) soils. For each soil, eight different colours are used, each representing a different level of synthetic spike (1–8, Table 1)

The experimental data (Fig. 3, symbols) is similar to the modelled set (Fig. 3, lines), indicating that despite a 100-fold difference in spike concentration, microbiota abundance can be accurately estimated. At every level of synthetic spikes, there were more sequenced reads per 1000 total reads for the F synthetic spike (ITS) than for the E synthetic spike (18S rRNA), which in turn, had more sequenced reads than the P synthetic spike (16S rRNA) (Fig. 3). As expected, prokaryotic 16S rRNA is more abundant than eukaryotic 18S rRNA gene, which in turn is more abundant than fungal ITS in soil. Furthermore, adding synthetic spikes (levels 1–8, Table 1) did not cause a significant change in the measured composition of the prokaryotic, eukaryotic and fungal communities (Fig. 4a–c). Data points for two eukaryotic samples showing an over-representation of Annelida DNA (with relative contribution of 25 and 70% compared to < 1% for other samples) were removed from Fig. 4 for clarity. These two soil samples were probably contaminated with a piece of earthworm. However, the size of the tissue was not large enough to alter the amount of detected 16S rRNA and ITS or their phylogenetic profiles.

### Determining domain abundance in two contrasting soils

**Fig. 5:**
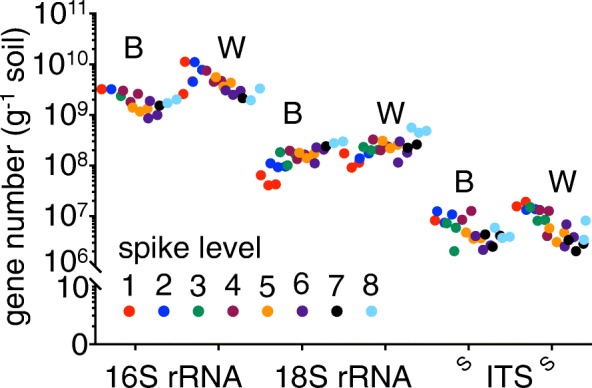
Estimated abundance of in situ microbial genes in Bawburgh (B) and Wytham (W) soils using synthetic spikes. Abundance in soil was calculated as follows: microbial rRNA abundance = (number of microbial-origin reads/number of synthetic-origin reads) × synthetic spike copies added to sample prior to DNA isolation for 16S rRNA with P synthetic spike, 18S rRNA with E synthetic spike and ITS with F synthetic spike. Gene copy number per gram soil on the *y*-axis is plotted against synthetic spike level (1–8, Table 1) on the *x*-axis

As the amount of synthetic spike DNA added to the soil is known, the in situ abundance in soil of each respective gene (16S rRNA, 18S rRNA or ITS) can be calculated (Fig. 5). For each synthetic spike level, there was relatively more 16S rRNA, 18S rRNA and ITS genes in Wytham soil compared with Bawburgh soil.

With a 100-fold concentration of spikes, there was a 4-, 6- and 7-fold variation in the total in situ abundance measured for 16S rRNA, 18S rRNA and ITS, respectively (Fig. 5). Omitting samples with less than 200 and more than 800 spike reads per 1000 total reads (dotted green lines on Fig. 3) (i.e. very low or high synthetic spike levels) reduced the variation to approx. 1.2- to 1.3-fold in each case (Fig. 5). Samples that fulfil these criteria are for 16S rRNA levels 7 and 8; for 18S rRNA levels 3, 4, 5 and 6; and for ITS level 1 and 2 (Table 2).

**Table 2:** Effect of spike level on estimated copy number of rRNA genes

Fraction	Bawburgh		Wytham	
	Mean	SEM	Mean	SEM
16S rRNA all	1.9E + 09	2.5E + 08	4.1E + 09	7.2E + 08
16S rRNA 200–800	1.6E + 09	2.3E + 08	2.2E + 09	2.3E + 08
18S rRNA all	1.5E + 08	2.4E + 07	2.2E + 08	3.6E + 07
18S rRNA 200–800	1.5E + 08	5.6E + 06	2.1E + 08	1.4E + 07
ITS all	5.6E + 06	9.8E + 05	7.7E + 06	1.7E + 06
ITS 200–800	8.3E + 06	8.9E + 05	1.4E + 07	1.7E + 06

Copies of the target gene are shown calculated from samples with addition of synthetic spikes at levels 1–8 (Table 1) (all) and for those which fulfil the criteria > 200 and < 800 synthetic spike reads per 1000 total reads (200–800). For 16S rRNA, gene 200–800 zone is levels 7 and 8, for 18S rRNA is levels 3,4, 5 and 6 and for ITS is levels 1 and 2

Using only samples fulfilling these criteria (Table 2), the estimation of abundance of prokaryotic 16S rRNA was reduced from 1.9 × 10^9^ to 1.8 × 10^9^ for Bawburgh soil and from 4.9 × 10^9^ to 2.5 × 10^9^ for Wytham soil. Eukaryotic 18S rRNA gene abundance remained almost identical at 1.5–1.7 × 10^7^, and 2.5–2.3 × 10^8^ while fungal ITS was increased from 5.8 × 10^6^ and 8.0 × 10^6^ to 9.8 × 10^6^ and 1.6 × 10^7^ for Bawburgh and Wytham soils, respectively. Fungal spike levels 3–8 were saturated with synthetic spikes and under-represented in situ microbial ITS abundance (Table 2). The requirement to tailor synthetic spike levels depending on the amplification target (i.e. 16S rRNA, 18S rRNA or ITS) correlates with the total abundance of in situ microbial DNA from each of these groups. We suggest using a concentration of synthetic spike at a level > 20% of the (expected) environmental microbial gene abundance. Oversaturation with synthetic spikes (> 80%) increases the sequencing depth necessary and may also bias results. In each case, the microbiota is more abundant in Wytham soil than in Bawburgh soil, showing 25, 29 and 40% more prokaryotes, eukaryotes and fungi, respectively. These results correlate with the fact that Wytham soil is richer in organic matter and macronutrients than that from Bawburgh.

We observe a gradient in microbiota abundance estimation based on the P and F spike level addition. Generally, the more spikes were added the lower microbiota abundance was recorded. This relationship was not observed for the 18S rRNA as well as for the 16S rRNA and ITS datasets inside the 20–80% ratio zone (Fig. 5). It suggests that the low levels of spikes may be retained in soil and hence soil type may cause a significant bias in the microbiota estimation, while very high levels oversaturate the DNA pool.

### Phylogenetic structure of the microbiota is altered by absolute quantitation

**Fig. 6:**
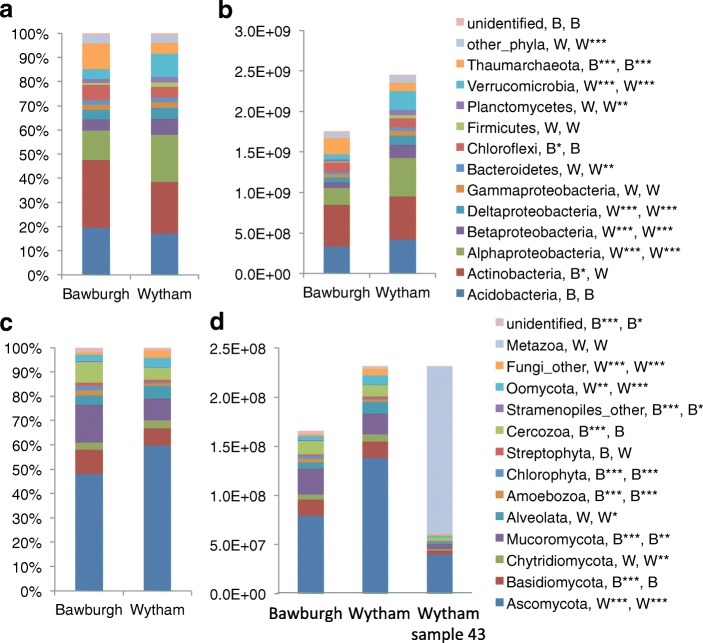
Prokaryotic and eukaryotic community structure in Bawburgh and Wytham soils**. a** Relative prokaryotic 16S rRNA gene abundance. **b** Absolute prokaryotic 16S rRNA gene abundance. **c** Relative eukaryotic 18S rRNA gene abundance and **d** absolute eukaryotic 18S rRNA gene abundance, an additional sample of Wytham soil is presented; ‘Wytham_43’ with a high proportion of macroorganism (Annelida) tissue present. Reads with Bit-score lower than 300 are clustered into ‘low-blast’ group. ‘B’ and ‘W’ indicate that the taxa in more abundant in Bawburgh or Wytham soil, respectively. *< 0.05, **< 0.01, ***< 0.001 using *t* test and Bonferroni correction for testing multiple comparisons

**Fig. 7:**
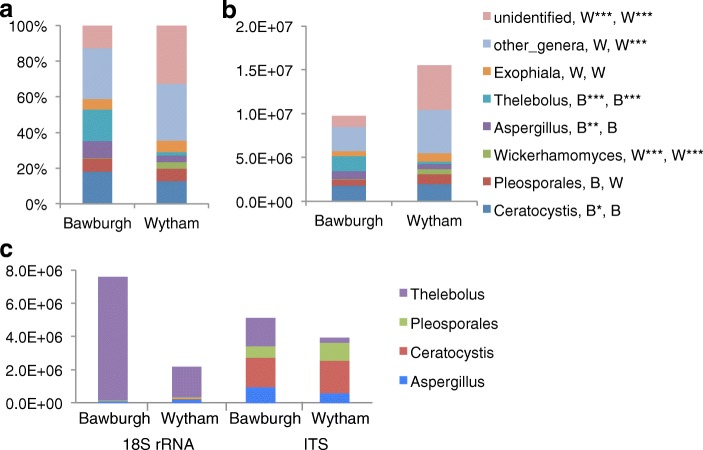
Fungal community structure in Bawburgh and Wytham soils**. a** Relative fungal ITS abundance. **b** Absolute fungal ITS abundance shown at the genus level. **c** Relative fungal ITS abundance at the divisional level. **d** Absolute fungal ITS abundance at the division level. **e** Comparative analysis based on 18S rRNA or ITS for absolute abundance of selected fungal genera. Reads with Bit-score lower than 300 are clustered into ‘low-blast’ group. ‘B’ and ‘W’ indicate that the taxa in more abundant in Bawburgh or Wytham soil, respectively. *< 0.05, **< 0.01, ***< 0.001 using *t* test and Bonferroni correction for testing multiple comparisons

Microbial phylogenetic profiles were constructed for soil samples from Bawburgh and Wytham based on prokaryotic 16S rRNA, eukaryotic 18S rRNA and fungal ITS sequences (Fig. 5). There is greater abundance of prokaryotes in Wytham soil (approx. 2.5 × 10^9^) compared to Bawburgh samples (approx. 1.8 × 10^9^). The relative (Fig. 6a, c and Fig. 7a, c) and quantitative results for the microbiota structures were compared (Fig. 6b, d and Fig. 7b, d). Proteobacteria, Actinobacteria and Acidobacteria are dominant phyla in Bawburgh and Wytham soils (Fig. 6a, b). This prokaryotic profile is commonly found in medium pH soils [30]. Using a relative approach (Fig. 6a), the main differences between these soils are higher abundance of Actinobacteria, Chloroflexi and Thaumarchaeota in Bawburgh soil and Alpha-, Beta-, Delta-proteobacteria, Planctomycetes and Verrucomicrobia in that from Wytham. However, when the quantitative correction was applied (Fig.6b), Bawburgh soil is no longer enriched with any phyla, apart from the Thaumarchaeota relative to Wytham, while Wytham is additionally enriched with Bacteroidetes. This is consistent with Wytham soil being richer than Bawburgh with a greater abundance of most groups.

Fungi, especially Ascomycota, Basidiomycota and Mucoromycota, dominate the eukaryotic community in both Bawburgh and Wytham soils. Quantitation indicates that the dominant phyla of Ascomycota and Chytridiomycota are more abundant in Wytham soil, while Mucoromycota is prevalent in that from Bawburgh (Fig. 6c, d). One of the soil samples (Wytham_43, Fig. 6d) contained a piece of Annelida tissue with more than 70% of the sequencing reads belonging to this group. As might be expected, a substantial piece of animal tissue increased the abundance of 18S rRNA copies (approx. 3.2 × 10^8^) over that of the average (approx. 2.5 × 10^8^) for Wytham soil.

### Insight into primers specificity

There is a clear difference between phylogenetic profiles and microbiota quantitation obtained using 18S rRNA and ITS primers. Fungi consist of 77% (corresponding to 1.3 × 10^8^ gene copies per 1 g of soil) and 79% (corresponding to 1.8 × 10^8^ gene copies per 1 g of soil) of the total 18S rRNA-based eukaryotic community for Bawburgh and Wytham soils, respectively. However, ITS-based analysis indicates that fungal abundance is much lower than the results suggested from the 18S rRNA analysis with values of 8.3 × 10^6^ and 1.4 × 10^7^ for these soils, respectively. 18S rRNA primers over-represent the abundance of fungal species and/or ITS primers do not capture the whole fungal taxonomy or both of these approaches produce a bias. Not surprisingly ITS and 18S rRNA-based analyses indicate a different absolute and relative abundance of common fungal genera (Fig. 7e). It is known that 18S rRNA primers are not able to unravel detail in fungal taxonomy; however, results from both primer sets agree on soil to soil comparison where *Ceratocystis*, Pleosporales and *Aspergillus* are more abundant in Wytham soil, while Thelebolus is more abundant in Bawburgh soil.

### Cross-domain comparison

**Fig. 8:**
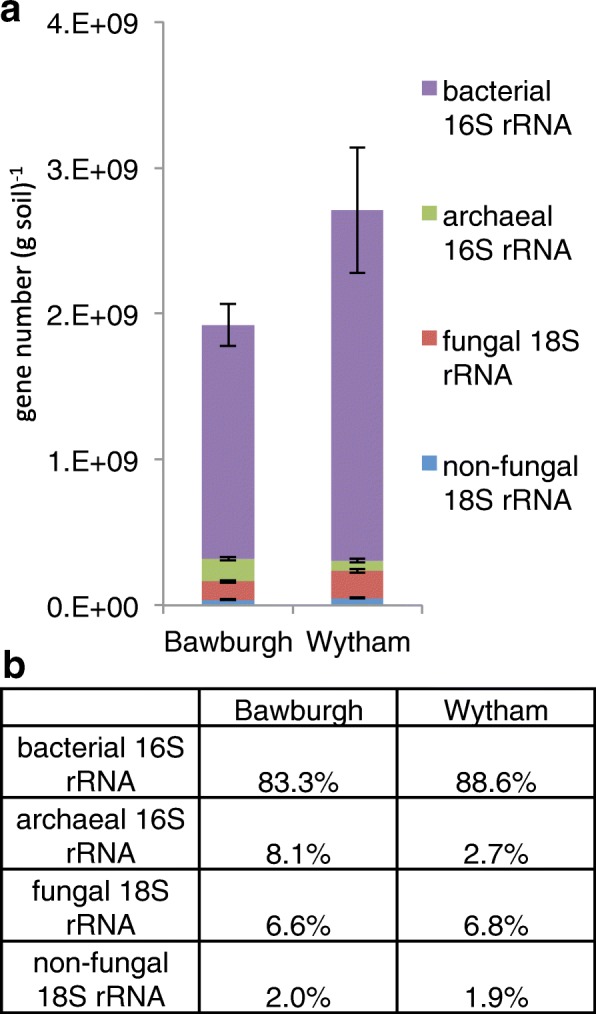
Comparison of absolute and relative microbiota abundance in soil. **a** Absolute bacterial and archaeal 16S rRNA and fungal and non-fungal 18S rRNA gene abundance and **b** their relative abundance

Amplification reactions using different primer pairs may not target a full range of potential microbial taxa. The 16S rRNA primers used may not target all prokaryotic species, and 18S rRNA primers may not target all eukaryotic taxa. However, assuming that these primers capture most of the community, the ratio of prokaryotic to eukaryotic ribosomal content can be determined (Fig. 8). Prokaryotes make up 91.4% and eukaryotes make up 8.6% of the soil microbiota. The limitation of our approach is that we cannot easily compare the results with microscope-based counts as prokaryotic cells normally have only a few ribosomal operons [31–33], while eukaryotes may have hundreds of copies [34–36]. Wytham soil is richer in both 16S rRNA and 18S rRNA relative to that from Bawburgh; however, the ratio is similar for both soils (Fig. 8). This potentially represents a biological mechanism controlling inter-domain relationships in (fallow) soils.

In order to confirm our findings, we have used qPCR on the DNA isolated from Bawburgh soil. By comparing the relative amplification efficiency of the 16S and 18S rRNA genes targets, we have calculated that eukaryotes contribute to 7.6% (max. 17%, min. 4%) and prokaryotes to 92.4% (max. 96%, min. 83%) of the soil microbial community (Additional file 6). These values are only 1% different from our spike-based approach results.

Our values can also be compared with results published from a meta-transcriptomic study, which showed that prokaryotic 16S rRNA and eukaryotic 18S rRNA contribute towards 91.5 and 2.8% of a soil microbiota (the remaining 6.1% of RNA detected could not be assigned) [27]. A study comparing soils of contrasting fungi to bacteria ratio found that fungi contributed 0.6 to 4.2% depending on the soil and detection method e.g. fatty acid analysis, RNA-seq and meta-proteomics [37]. The ratio of fungi to bacteria in our study is higher, 8.0% (fungi contribute 6.6% and bacteria contribute 83.3% for the total microbiota) and 7.7% (fungi contribute 6.8% and bacteria contribute 88.6% for the total microbiota) for Bawburgh and Wytham soils, respectively (Fig. 8). An elevated ratio of fungi to bacteria has been associated with high soil fertility, high levels of organic carbon and lack of tillage [38]. The fallow soils used in this study may indeed have a high fungal abundance, as their organic content is high and they have not been tilled for decades.

## Discussion

Absolute quantitation of the microbiota is essential for all aspects of microbial ecology, and our approach accurately estimated bacterial culture and soil microbiota numbers. The number of synthetic spikes added to soil over several orders of magnitude was directly proportional to the number of sequence reads obtained. While variation between replicates was usually low, some samples had larger errors, confirming that with very complex samples, such as soil, high numbers of replicates are advisable. For example, in previous work investigating the plant-soil microbiota, we used twenty-four biological replicates for each condition [24]. In this work, due to the need to test a large gradient of synthetic spike levels, we reduced the number to three replicates for each of the eight synthetic spike levels. However, ideally for critical analysis of the soil microbiota, replicate numbers should be greater than this. Prokaryotic 16S rRNA was detected at around 10^9^ and eukaryotic 18S rRNA at around 10^8^ copies per gram of soil; however, there may be up to an order of magnitude difference in contrasting soils [38]. For example, an extensive study of two Chinese soils estimated bacterial load (bacterial cells per 1 g of soil) at 8 × 10^8^ and 1.9 × 10^8^ based on ATP concentration, 1.7 × 10^9^ and 2.5 × 10^8^ based on flow cytometry, 6.2 × 10^9^ and 1.6 × 10^9^ based on qPCR, 1.3 × 10^9^ and 8.1 × 10^8^ based on phospholipid-derived fatty acid and 1.4 × 10^10^ and 1.0 × 10^10^ based on minimum bactericidal concentration for rich Beijing and poor Tibetan soil, respectively [13]. Using the van Bammelen factor conversion (0.58 × soil organic matter = soil organic carbon), we can compare total soil organic carbon content between these Chinese and our soils: in mg/g of soil; Beijing 21.5, Wytham 9.73, Tibetan 1.50, Bawburgh 1.69). Our values of 1.8 × 10^9^ and 2.5 × 10^9^ of 16S and 18S gene copies per g of soil, for the Bawburgh and Wytham, respectively, are similar to the above microbiota abundance values described for the Chinese soils.

Wytham soil, which is rich in organic matter, showed higher microbial ribosome abundance than poorer Bawburgh soil. This is true both for prokaryotic and eukaryotic microbiotas (Fig. 5). Quantitation allows for statistical correction of soil microbial abundance. Many microbial taxa are actually more abundant in Wytham soil even though their relative presence is higher in Bawburgh (Figs. 6 and 7). We believe this to be fundamentally important for all aspects of microbiota research and ecology, and it applies to microbiotas from all environments, from soil to the mammalian gut. Without absolute quantitation of groups, the underlying physiology and ecology of the role of specific microbial taxa may be masked by their relative abundance. For example, changes in the absolute abundance of keystone symbionts or pathogens may be masked by unaltered relative abundance or the relative abundance may go in the opposite direction to absolute abundance. This is likely to be because the relative abundance of any specific group is highly dependent on the absolute abundance of the most numerous organisms.

Since the experimental setup allows detection of the majority of microbial taxa, i.e. 16S rRNA and 18S rRNA primers targeting most of the prokaryotic and eukaryotic diversity, we were able to compare abundance of these domains of life (Fig. 8). These results agree with previous RNA-based estimates of the soil microbiota [27]. Adding synthetic spikes allowed accurate detection of microbial 16S rRNA presence in control samples of a *Rhizobium* culture, with it being more effective to add spikes directly to environmental samples rather than to isolated DNA (Fig. 2).

DNA spiking may be combined with other pre-processing steps, for instance with the removal of relic DNA (extracellular DNA). Propidium monoazide dye reacts with DNA not protected by a cellular membrane and subsequently blocks its PCR amplification [39]. Synthetic spikes could be added to aliquots of the initial sample and that with relic DNA removed. This approach would reveal which taxa are alive and their absolute presence.

It is strongly recommended that an initial calibration curve is performed to determine the optimal synthetic spike level for a given environmental condition. Spike addition in too high or too low, an amount compared to the targeted microbiota, may skew the quantitative results although the level of synthetic spike used did not alter the measured structure of the microbial community (Figs. 4, 5, and 6). However, once an initial calibration is performed, the level of spike could be varied over three orders of magnitude with high reproducibility. Care needs to be taken to ensure efficient isolation of DNA and its stabilisation from different environments as this can bias any method of quantification. However, given this caveat, our approach is simple, requiring only addition of known amounts of synthetic spike DNA and a single bioinformatic step post-sequencing, in order to quantify the absolute abundance of prokaryotes, eukaryotes and fungi in microbiota studies.

## Conclusion

Quantification of the active microbiota will contribute to a better understanding of functional groups in environmental microbiology and can help in producing better microbiota interactions models [40]. Such quantification has widespread application to microbiota/metagenome-wide association studies linked to disease [41] or soil productivity [42].

## Additional files

Additional file 1: Experiment preparation details: spike sequences, primers sequences, Rhizobium experiment spike output and spike concentration to numbers calculation. (XLSX 17 kb)

Additional file 2: Length of the sequences obtained for Bawburgh vs. Wytham soil comparison. (PDF 133 kb)

Additional file 3: Data used for figures and tables preparation. (XLSX 54 kb)

Additional file 4: **Figure S1.** Model of synthetic spike addition. Number of sequencing reads of synthetic spike per 1000 total reads (*y* axis) from 16 s rRNA, 18S rRNA and ITS following addition to soil of different levels of P, E and F synthetic spikes (*x* axis). The figure presents the full dataset from the averaged dataset shown on Fig. 3. (TXT 39 kb)

Additional file 5: **Figure S2.** Estimated abundance of in situ microbial genes in Bawburgh (B) and Wytham (W) soils using synthetic spikes. The figure presents the full dataset from the averaged dataset shown on Fig. 5. (PDF 84 kb)

Additional file 6: qPCR data. (TXT 12 kb)

Additional file 7: Description of samples submitted to EBI server. (XLSX 74 kb)

Additional file 8: Bash code for the sequencing analysis. (XLSX 63 kb)

Additional file 9: Modified Bash code including Unoise3 command. (XLSX 422 kb)

## Abbreviations

16S and 18S rRNA: 16S and 18S ribosomal RNA genes
ITS: Internal transcribed spacer
MDS: Multidimensional scaling plot
OTU: Operational taxonomic unit
P, E and F spikes: Prokaryotic, eukaryotic, and fungal specific spikes
PBS: Primer binding site
PCR: Polymerase chain reaction
TY: Tryptone yeast extract medium
