# Supplementary figures and images for: Absolute quantitation of microbiota abundance in environmental samples
Source: Microbiome. 2018 Jun 19;6:110. doi: 10.1186/s40168-018-0491-7 (PMC6009823; doi:10.1186/s40168-018-0491-7)

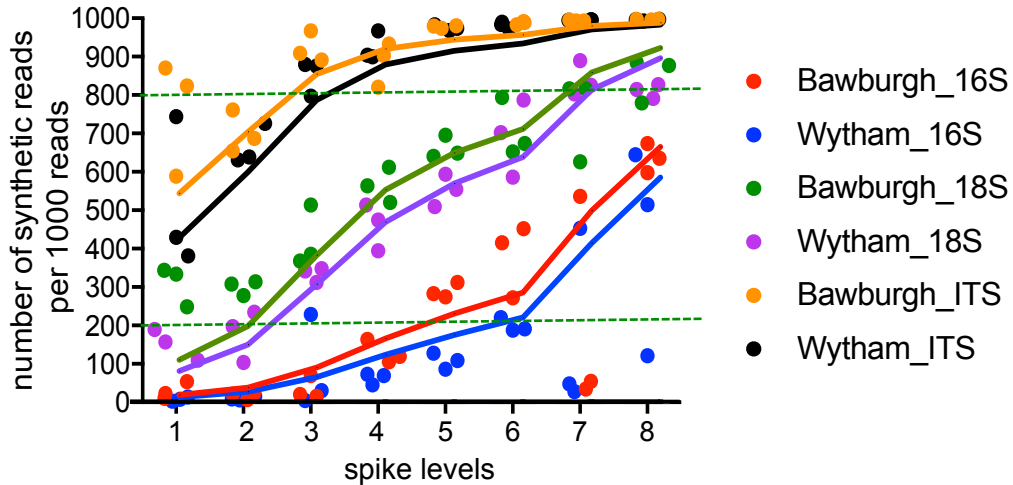

Supplement: Supplementary file 2 — Length of the sequences obtained for Bawburgh vs. Wytham soil comparison. (PDF 133 kb) [file 40168_2018_491_MOESM2_ESM.pdf]

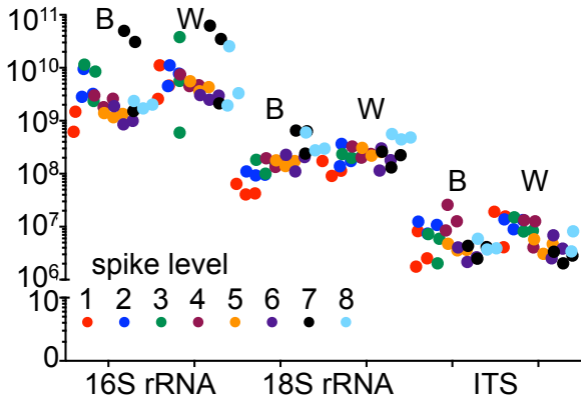

Supplement: Supplementary file 5 — Figure S2. Estimated abundance of in situ microbial genes in Bawburgh (B) and Wytham (W) soils using synthetic spikes. The figure presents the full dataset from the averaged dataset shown on Fig. 5. (PDF 84 kb) [file 40168_2018_491_MOESM5_ESM.pdf]
